# Left ventricular assist device utilization across the different regions of the Netherlands

**DOI:** 10.1007/s12471-026-02019-9

**Published:** 2026-02-10

**Authors:** Valérie C. E. Drost, Maaike Wösten, Luuk C. Otterspoor, Kevin Damman, Laurens F. Tops, Monica Gianoli, Michiel Kuijpers, Meindert Palmen, Jelena Sjatskig, Aria P. Yazdanbakhsh, Kadir Caliskan, Linda W. Van Laake

**Affiliations:** 1 Thoraxcenter, Department of Cardiology, Cardiovascular Institute, Erasmus MC University Medical Center, Rotterdam, The Netherlands; 2 Thoraxcenter, Department of Cardiothoracic Surgery, Erasmus MC University Medical Center, Rotterdam, The Netherlands; 3 Department of Cardiology, Catharina Hospital, Eindhoven, The Netherlands; 4 Department of Biomedical Engineering, Eindhoven University of Technology, Eindhoven, The Netherlands; 5 Department of Cardiology and Transplantation Center Utrecht, University Medical Center Utrecht, Utrecht, The Netherlands; 6 Computational Imaging Group for MRI Therapy & Diagnostics, University Medical Center Utrecht, Utrecht, The Netherlands; 7 Department of Cardiothoracic Surgery, University Medical Center Utrecht, Utrecht, The Netherlands; 8 Utrecht University, Utrecht, The Netherlands; 9 Department of Cardiology, University Medical Center Groningen, University of Groningen, Groningen, The Netherlands; 10 Department of Cardiology, Leiden University Medical Center, Leiden, The Netherlands; 11 Department of Cardiothoracic Surgery, Leiden University Medical Center, Leiden, The Netherlands; 12 Department of Cardiology, Onze Lieve Vrouwe Gasthuis, Amsterdam, The Netherlands

**Keywords:** Heart Failure, Left Ventricular Assist Device, Healthcare Utilization, Healthcare Disparities, Socioeconomic Status

## Abstract

**Introduction:**

The accessibility of left ventricular assist device (LVAD) therapy is a crucial factor in the survival and quality of life of patients suffering from advanced heart failure. However, there is a lack of clarity regarding the utilization of this therapy across regions in the Netherlands as well as whether any disparities exist based on socioeconomic status (SES). This study aimed to determine the utilization of LVAD therapy at a regional level using administrative data and to identify potential disparities based on SES by comparing postal code data to demographic governmental data.

**Methods:**

All patients aged 16 or older who underwent a primary LVAD implantation between 2015 and 2024 were included. The data was visualized with a heatmap using Python.

**Results:**

A total of 710 patients received an LVAD during the study period. LVAD utilization was lower in the southernmost regions compared to the northernmost regions and varied in the central regions. An ANOVA test between SES groups did not show significant differences in LVAD utilization (*p* = 0.20).

**Conclusion:**

The findings of this study indicate that there are notable variations in the utilization of LVAD therapy across different geographical regions in the Netherlands. Nevertheless, no differences in LVAD use were found between areas with different SES categories. Future research should focus on identifying the underlying factors associated with referral for advanced heart failure therapies to ensure equitable access to LVAD therapy.

**Supplementary Information:**

De online versie van dit artikel (10.1007/s12471-026-02019-9) bevat aanvullend materiaal, toegankelijk voor daartoe geautoriseerde gebruikers.

## What’s new?

Left ventricular assist device utilization across the different regions of the Netherlands

- Differences exist in the utilization of LVADs across different regions of the Netherlands among adult patients who received primary implantation during the study period (2015–2024).
- No disparities were identified in LVAD utilization between different socioeconomic status categories.
- Future research should focus on identifying factors associated with referral for advanced heart failure therapies.

## Introduction

Over the past two decades, left ventricular assist device (LVAD) implantation has gained acceptance as a therapy for patients suffering from advanced heart failure [1]. Due to technical innovations and improved quality of multidisciplinary management, LVAD therapy is now used as a bridge to heart transplantation, a bridge to candidacy or decision, or as destination therapy, offering an appropriate alternative for those ineligible for heart transplantation [2]. Continuous-flow, fully magnetically levitated LVADs are now the mainstay of therapy, and the outcome for these patients continues to improve with a one-year survival rate of 83% [3]. The pivotal MOMENTUM 3 randomized trial reported a five-year survival rate of 58% for patients using the Heartmate 3 [4]. More recent European data report a similar five-year survival rate of 54–63%, proving LVAD therapy to be a life-saving and quality-of-life-enhancing treatment for patients with advanced heart failure [5, 6].

Since the implementation of LVAD therapy as destination therapy in the Netherlands in 2015, an increasing number of patients have undergone LVAD implantation. Between 2016 and 2020, the number of implantations increased from 73 to 106 per year [7]. Yet, LVAD utilization remains limited in the context of heart failure being the fifth most frequent cause of death in the Netherlands [8]. However, little is known about the utilization of LVAD therapy at a regional level. The Netherlands has four LVAD implantation centers, which operate in a ‘hub and spoke’ configuration with referring secondary care centers [9]. Understanding the regional distribution of LVAD recipients in the Netherlands is essential to evaluate access to LVAD care and for identifying potential disparities. This is relevant, since identification and referral to advanced heart failure centers is often delayed and continues to be a critical challenge [10, 11]. In this light, the number of LVAD implantations may indirectly reflect referral for advanced heart failure therapies in general.

Easy access to heart failure therapy is critical to prevent high mortality rates and improve health outcomes, especially for patients with advanced heart failure [9]. Demographic factors such as socioeconomic status (SES) may influence access to LVAD therapy. Previous research has shown that lower SES is associated with a lack of specialist referral, a higher risk of morbidity and mortality in patients with heart failure, and lower use of cardiac resynchronization therapy (CRT) and the number of implantable cardioverter defibrillator (ICD) implantations [12]. However, to date, no studies have focused on the effect of SES on LVAD implantation rates in the Netherlands.

The primary objective of this study was to visualize the utilization of LVAD therapy in different postal code areas in the Netherlands. The secondary objective was to describe LVAD utilization at the postal code level, in relation to population characteristics, including SES and prevalence of cardiovascular mortality. Insights from this study may contribute to the future development of strategies to promote access to advanced heart failure care, including increased awareness of LVAD therapy.

## Methods

A retrospective multicenter study was conducted utilizing anonymous administrative data from patients who underwent LVAD implantation at one of the four Dutch implantation centers (Erasmus Medical Center Rotterdam, Leiden University Medical Center, University Medical Center Groningen, and University Medical Center Utrecht) in the Netherlands as enrolled in the European Registry for Patients with Mechanical Circulatory Support (EUROMACS) [13]. In addition, cardiovascular mortality and SES data were obtained from the open-access database of Statistics Netherlands (CBS) [14, 15].

### Study population

All consecutive patients aged 16 years and older who underwent LVAD implantation between January 2015, and January 2024, in one of the four centers were included. Furthermore, only primary implantations of patients who were both residing and implanted in the Netherlands were included.

### Data collection

Residential data of all patients who were implanted with an LVAD between 2015 and 2024 were collected from the administrative database of participating centers. For privacy purposes, residential data were categorized by 2‑digit postal code areas. Open-access, population-based mortality data due to cardiovascular disease of 2019 were collected from StatLine at the provincial level [14]. Furthermore, open access, population-based SES data of 2019 were collected from CBS at the 4‑digit postal code level [15]. CBS utilizes the SES-WOA score to determine SES. SES-WOA (Welvaart, Opleidingsniveau, recent Arbeidsverleden) is a composite score that integrates welfare, education level, and employment record. Higher SES-WOA scores indicate a higher SES classification [16]. SES-WOA scores for the 2‑digit postal code areas were calculated as the median of the SES-WOA scores of the corresponding 4‑digit postal code areas. In case of missing values, the SES-WOA score of the 4‑digit postal code area was replaced with the median of the corresponding 2‑digit postal code area. Data from 2019 was used for both the SES-WOA scores and cardiovascular mortality, since this year is the midpoint within the data collection period and before the COVID-19 pandemic, which may have influenced cardiovascular mortality especially.

### Ethics statement

This retrospective multicenter study used anonymous administrative data as well as open government data and did not involve direct participation of human subjects.

### Outcomes

The primary outcome was the number of patients implanted with an LVAD per 1 mio. population in each 2‑digit postal code area. The secondary outcome was the frequency of LVAD implantation per postal code area, stratified by SES.

### Data analysis

To illustrate the number of LVAD implantations per 1 mio. population per postal code area, a heatmap was created using Python (version 3.12, Wilmington, Delaware, USA). SES-WOA scores were divided into four quartiles, with SES1 being the lowest and SES4 being the highest. LVAD utilization relative to population size was compared between all four SES quartiles using one-way ANOVA. A *p*-value of 0.05 was considered statistically significant. Statistical analysis was performed using Python.

## Results

**Fig. 1:**
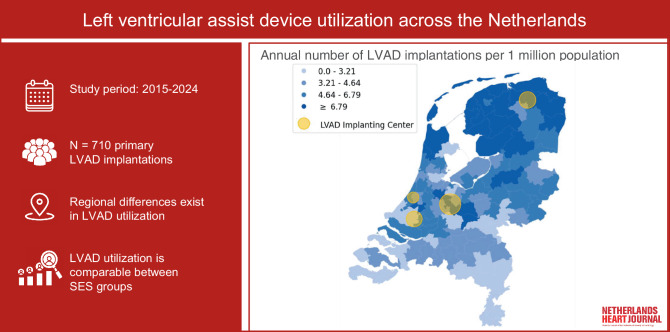
Annual number of implantations per 1 mio. population for each 2‑digit postal code area in the Netherlands. Implanting centers are indicated in yellow, with marker size corresponding to the number of implantations per center

Cardiovascular diseases as a cause of death in 2019 were highest in the provinces Drenthe, Limburg, and Zeeland, while it was the lowest in Noord-Holland, Flevoland, and Utrecht according to the data from the CBS. Figure 1a shows a heatmap of cardiovascular mortality in the Netherlands, with a darker red indicating higher rates. Figure 1b shows the SES-WOA score distribution in the Netherlands, demonstrating the four quartiles of the 2‑digit postal code SES-WOA scores (SES1: −0.33–−0.03; SES2: −0.03–0.06; SES3: 0.06–0.11; SES4: 0.11–0.21).

### LVAD implantations

During the study period of 9 years, 710 patients in the Netherlands were implanted with an LVAD with a mean of 79 implantations per year. This corresponds with 4.6 implantations per 1 mio. population annually.

**Fig. 2:**
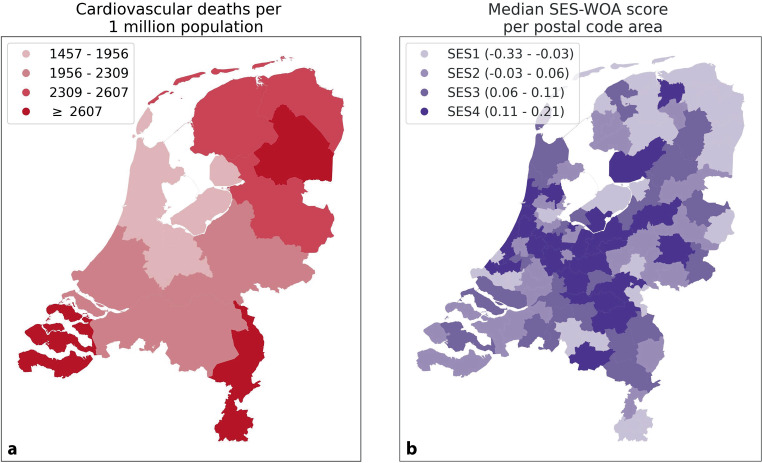
**a** Cardiovascular deaths per 1 mio. population in 2019 per provincial area in the Netherlands [12] and **b** Median SES-WOA score per 2‑digit postal code area in the Netherlands. SES-WOA: Socioeconomic Status—Welfare, Educational Level, Employment History [13]

Figure 2 depicts a map of the Netherlands with the number of LVAD implantations illustrated in quartile bins (0.0–3.21; 3.21–4.64; 4.64–6.79; ≥ 6.79 per 1 mio. population). Implantation frequency was the highest in the northern regions, varied in the central regions, and was lowest in the southern regions. In yellow, the four implantation centers are indicated; their size corresponds to the annual number of implantations.

An additional analysis was performed to study the effect of the distance between the 2‑digit postal code area of the patient and the implanting center. For this analysis, the straight line distance between each 2‑digit postal code area and the 2‑digit postal code area of the nearest implantation center was calculated. Subsequently, the correlation between the distance and implantation frequency was calculated. This resulted in a Pearson correlation coefficient of −0.13 and a *p*-value of 0.21.

**Fig. 3:**
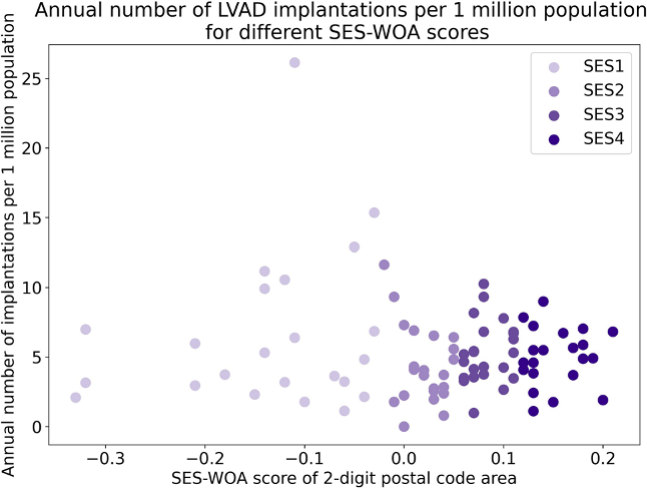
Annual number of LVAD implantations per 1 mio. population for different socioeconomic status scores (*p* = 0.20)

Figure 3 demonstrates the distribution of the median SES-WOA scores at corresponding LVAD implantation frequency per region. SES1, the lightest purple, represents a SES-WOA score between −0.03 and −0.33, while SES4 in the darkest color, represents a SES-WOA score between 0.11 and 0.21. One-way ANOVA resulted in a *p*-value of 0.20, showing that in this sample, there was no significant difference in LVAD implantation between the four SES categories (*Table S1*).

## Discussion

In this descriptive study, we visualized the regional distribution of LVAD implantations in the Netherlands from 2015 to 2024. Also, we compared implantation rates between different levels of SES. We noted differences in LVAD utilization between postal code areas, with a higher frequency of LVAD implantation per 1 mio. population in northern and central regions. Additionally, this study demonstrates that there is no significant relation between distance to the implantation center and implantation frequency. Similar to the higher implantation rate, the cardiovascular mortality was also high in the northern regions. In contrast, areas in the southern parts of the country show substantially lower rates of LVAD implantation, while cardiovascular mortality was also high in southern provinces. Here, it should be mentioned that although related and therefore showing the distribution of cardiovascular diseases in the Netherlands, heart failure is only one of the cardiovascular diseases that can cause a high cardiovascular mortality.

The average annual rate of LVAD implantation in the Netherlands corresponds to 4.6 per 1 mio. population. As compared with a survey by the European Society of Cardiology, the Netherlands ranks among the countries in Europe with the highest implantation rates per 1 mio. population. Compared to its neighboring countries, the Netherlands ranks lower than Germany (13.9), but still higher than Belgium (4.1), Denmark (3.2), and the United Kingdom (0.6) [17]. This may be partly explained by the lack of reimbursement for destination therapy for LVADs in some of these countries and long waiting times for heart transplantation in the Netherlands, necessitating long-term LVAD therapy. Although the implantation rate in the Netherlands is high by European standards, there is room for improvement in the utilization of LVAD therapy, as it is expected that a proportion of patients with advanced heart failure, the fifth leading cause of death in the Netherlands for years, will be overlooked. The underutilization of LVAD therapy, a potentially life-saving therapy in these patients, has been previously described [18]. Strikingly, research using market data across European countries showed that the utilization of LVAD devices does not mirror the increase in heart failure mortality [19]. Therefore, raising awareness for recognition of advanced heart failure and timely referrals remains critical.

Low SES is associated with an increased incidence of heart failure, related adverse events, and mortality [20]. Moreover, research shows that lower SES is also associated with lower utilization of potentially life-saving device therapy, including CRT and ICD [12]. However, studies have not yet assessed the influence of SES on LVAD utilization. In our study, no significant differences were observed in LVAD utilization between areas in different SES categories. These results suggest that the use of LVAD therapy in the Netherlands was not influenced by SES. Nevertheless, it is unknown what the need for LVAD therapy is for different SES scores. This need could be different over the different SES groups, for example, due to the association of low SES with increased incidence of heart failure and differences in therapy, possibly causing an underestimation of the effect of SES on implantation frequency [12, 20].

The observed geographic differences in LVAD utilization may be explained by several other factors, such as the level of awareness of referral criteria among physicians and ability to identify optimal timing of referral to advanced heart failure centers. Physicians have been found to overlook potential LVAD candidates due to older age or comorbidities and are still adhering to previously strict criteria for heart transplantation [21]. Timely referral is a critical factor in improving outcomes for advanced heart failure patients who may benefit from LVAD therapy. Particularly for relatively younger heart failure patients aged < 70 years, who were initially eligible candidates, the disease progression and comorbidities can make a patient ineligible for both transplantation and LVAD therapy [9, 22]. LVAD utilization could also depend on the patient’s awareness of available advanced HF treatment options. Patients generally rely on their treating physician for this information [23]. Furthermore, physicians should have sufficient clinical confidence in LVAD therapy in eligible patients [10]. Therefore, understanding the awareness of referral criteria among health care providers and patients, along with reasons behind both referral and non-referral, could offer valuable context for regional utilization of LVAD therapy. In future research, temporal analysis of the annual implantation rate at the regional level can show changes over time. Such year-by-year information may provide actionable insights for improving access to LVAD therapy.

### Limitations

For interpretation of the results, it should be noted that in this study, the most recent available residential data was used to determine LVAD implantation frequency. Additionally, referrals to implanting centers outside the Netherlands were not taken into account, which could potentially lead to underestimation of the number of implantations. However, considering the healthcare reimbursement system in the Netherlands, this number is expected to be negligible.

Also, in this study, we used the median SES-WOA score per 2‑digit postal code area. Within these areas, differences in SES-WOA scores may exist at the individual level. For a more detailed analysis of the relation between individual LVAD implantation and SES-WOA score, household SES-WOA scores could be reviewed.

Furthermore, the observed regional utilization of LVAD therapy may also be impacted by several other related factors. These could be patient-related factors, such as heart failure incidence, corresponding age, comorbidities, and logistical factors such as mobility. Although detailed information about these topics was not available for analyses in this study, these factors are not likely to be reversely correlated to cardiovascular mortality and would therefore not explain the geographical inequity observed in our study. A general impression of baseline characteristics of patients implanted with an LVAD between 2016 and 2020 in the Netherlands is provided by Damman et al. [7]. Other factors that could have impacted LVAD utilization may be linked to physician awareness, referral patterns, and inter-hospital agreements and collaborations. An example of the latter can be the use of a shared care model as described by Drost et al. [24].

Overall, utilization of LVAD therapy may depend on many factors and results from a comprehensive interaction between the referring center, implanting center, and the patients’ needs and preferences, which are not all covered in the data presented in this study. Therefore, conclusions on causal relationships could not be drawn. The use of advanced heart failure data at the regional level would have enabled us to study the relation between advanced heart failure incidence and LVAD utilization. Unfortunately, this data was not available. To allow for such analyses, we recommend further improving the availability and completeness of detailed heart failure databases at the national level, such as the Netherlands Heart Registration and Heart4Data [25, 26].

## Conclusion

In conclusion, this study shows that there are regional differences in the use of LVAD therapy in the Netherlands. No differences in LVAD use were found between areas with different SES categories. Several other hypotheses may provide an explanation for the observed regional differences, such as lower recognition of patients meeting the criteria for advanced heart failure therapy and/or referral bias. Further research is needed to identify the underlying factors associated with the identification of patients with advanced heart failure and timely referral to potentially life-saving solutions for end-stage heart failure.

## Supplementary Information

*Tab. S1* Implantation frequency per socioeconomic status category between 2015 and 2024

## Data Availability

The data underlying this article will be shared on reasonable request to the corresponding author.
