# Supplementary material for: Left ventricular assist device utilization across the different regions of the Netherlands
Source: Neth Heart J. 2026 Feb 10;34(3):100–6. doi: 10.1007/s12471-026-02019-9 (PMC12920953; doi:10.1007/s12471-026-02019-9)
Supplement: Supplementary file 1 — Tab. S1 Implantation frequency per socioeconomic status category between 2015 and 2024 [file 12471_2026_2019_MOESM1_ESM.docx]

**Supplementary information**

**Table S1.** Implantation frequency per socioeconomic status category between 2015 and 2024

| **SES-WOA classification** | | **Annual number of implantations per 1.000.000 population** |
| --- | --- | --- |
|  | *Range of median SES-WOA score* | *Median [IQR]* |
| SES1 | (-0.33, -0.03) | 4.8 [3.1 – 8.5] |
| SES2 | (-0.03, 0.06) | 3.9 [2.4 – 6.2] |
| SES3 | (0.06, 0.11) | 4.9 [3.6 – 6.7] |
| SES4 | (0.11, 0.21) | 4.9 [3.8 – 6.7] |
